# Perceptions of healthcare professionals regarding the main challenges and barriers to effective hospital infection control in Mongolia: a qualitative study

**DOI:** 10.1186/1471-2334-12-170

**Published:** 2012-07-31

**Authors:** Bat-Erdene Ider, Jon Adams, Anthony Morton, Michael Whitby, Archie Clements

**Affiliations:** 1 University of Queensland, School of Population Health, Queensland, QLD 4006, Australia; 2 University of Technology Sydney, Faculty of Nursing, Midwifery and Health, New South Wales 2007, Australia; 3 Infection Management Services, Princess Alexandra Hospital, Queensland, QLD 4102, Australia; 4 Greenslopes Private Hospital, Queensland, QLD 4120, Australia; 5 University of Queensland, School of Population Health, Queensland, QLD 4006, Australia

**Keywords:** Infection control, Qualitative research, Challenges, Barriers, Mongolia

## Abstract

**Background:**

It is not fully understood why healthcare decision-makers of developing countries often give low priority to infection control and why they are unable to implement international guidelines. This study aimed to identify the main perceived challenges and barriers that hinder the effective implementation of infection control programmes in Mongolia.

**Methods:**

In 2008, qualitative research involving 4 group and 55 individual interviews was conducted in the capital city of Mongolia and two provincial centres.

**Results:**

A total of 87 health professionals participated in the study, including policy and hospital-level managers, doctors, nurses and infection control practitioners. Thematic analysis revealed a large number of perceived challenges and barriers to the formulation and implementation of infection control policy. These challenges and barriers were complex in nature and related to poor funding, suboptimal knowledge and attitudes, and inadequate management. The study results suggest that the availability of infection control policy and guidelines, and the provision of specific recommendations for low-resource settings, do not assure effective implementation of infection control programmes.

**Conclusions:**

The current infection control system in Mongolia is likely to remain ineffective unless the underlying barriers and challenges are adequately addressed. Multifaceted interventions with logistical, educational and management components that are specific to local circumstances need to be designed and implemented in Mongolia. The importance of international peer support is highlighted.

## Background

It has been widely known for the last four decades, that the majority of healthcare-associated infections (HCAIs) can be prevented by adequate, though not necessarily sophisticated, surveillance and control measures [1,2]. A number of international initiatives are being undertaken to support developing countries to build and implement infection control effectively in their health care settings [3-6]. Despite these growing efforts, infection control in most developing countries remains either non-existent or ineffective, posing a significant threat to quality of patient care [7-9]. In 2010, the WHO reported that only 23/147 developing countries have a functioning surveillance system for HCAI, which is a core part of infection control programs [10].

In Mongolia, the current HCAI prevention and control system was established in 1997. Management structures for HCAI prevention and control were implemented at national and hospital levels, infection control practitioners (ICP) were designated in hospitals and guidelines were provided on HCAI control [11]. Several international consultants have visited hospitals and advised the government to initiate surveillance for specific groups of HCAI alongside other strategies such as hand-hygiene promotion, training of hospital staff and improving laboratory capacity. In 2002, the Government approved a national programme to establish a sentinel surveillance system for surgical and infant infections with improved laboratory-based monitoring of multi-resistant organisms [12]. Although there were some advances in hand-hygiene and infection control policies and guidelines were updated, the planned surveillance activities have not yet been established.

For future planning and to provide insight for infection control in other developing countries, it is crucial to know what factors have restricted implementation of infection control policies and programmes in Mongolia. To date, there has been no research on reasons for infection control programme implementation failure in Mongolia, either published or unpublished, and there has been little such research conducted in other developing countries. This study aims to identify the perceptions of healthcare professionals on the main challenges and barriers that hinder the effective implementation of infection control programmes in Mongolia. The findings are interpreted to provide insights of benefit for policy makers and healthcare managers not only in Mongolia but also other developing countries.

## Methods

### Data collection

The study was approved by the ethics committees of the Ministry of Health (MoH) of Mongolia and the University of Queensland, Australia. In 2008, qualitative research involving semi-structured group and individual interviews was conducted in the capital city of Mongolia, Ulaanbaatar, and the capital cities of the Selenge and Dornogovi provinces. A purposive sampling method was used to recruit key informants from MoH, Health Related Infection Surveillance and Research Unit (HRISRU), district and province health services, tertiary hospitals and the State Inspection Agency (SIA) [13]. A supplementary snowballing technique added participants from the Health Insurance Department, the Health Sciences University and the Nursing College. Ninety-one health professionals were approached and asked to provide consent to participate; four of them refused due to time constraints. The principal investigator (B-E.I.) moderated both group discussions and interviews using the same semi-structured guide. The moderator acted as a guide for the participants helping to maintain the flow and level of discussion when relevant through general prompts and probes. However, both individual interviews and group discussions were organised as to encourage participants to introduce topics and issues that they perceived as relevant and important and to also discuss issues in their own words. The moderator was previously involved in health care quality and health service research projects and was known to some policy and hospital level senior managers. However, the moderator had no direct relationship with the participants and all roles were disclosed and cleared prior to commencing fieldwork so as to ensure the discussion and data was not unduly compromised or influenced. The 4 group discussions took 41–104 minutes and involved mainly nurses and ICPs. Participants who could not attend group discussions were also invited to attend individual interviews. The 55 in-depth interviews involved mainly administrative staff and doctors and took 14–74 minutes. Preliminary data analysis was carried out at this stage; emergent themes and issues from earlier interviews shaped the structure of subsequent discussions. The discussions were recorded digitally and ceased at a saturation point at which no new themes emerged.

### Data analysis

All audio files were entered into NVivo-8 software (QSR International, Melbourne, Australia). The principal investigator (B-E.I.) translated and transcribed the data. The five-step framework approach was used in the thematic analysis [14]. Following the data familiarisation process, the research team (B-E.I., A.C.A.C., J.A.) identified the major themes after a series of iterative discussions. Then B-E.I performed the coding by indexing the presence of each theme and selecting quotations. Any difficulties in the interpretation and categorization of data were resolved by team discussions. Data were triangulated (i.e. cross-referenced) between participant groups. The issues of gaming (fraudulent reporting, manipulation of data and excessive use of antibiotics to hide HCAIs) in infection control data was analysed separately and reported previously [15].

## Results

A total of 87 health professionals, including 42 health policy and hospital level managers, 6 doctors, 9 nurses, 35 infection control professionals and 8 other health professionals participated in the study (Table 1). Challenges and barriers to successful implementation of infection control programmes in Mongolia perceived by these study participants were grouped into those impinging on: (1) the formulation; and (2) the implementation of infection control policy.

**Table 1:** Characteristics of participants in interviews and focus group meetings regarding challenges and barriers to effective infection control in Mongolia, 2008 (N = 87)

**Basic attributes**	**Number (%)**
Gender	
Male	19 (22)
Female	68 (78)
Position	
Manager	37 (42)
Doctor	5 (6)
Nurse	8 (9)
ICP	24 (28)
IC team member (lab, sterilization unit)	6 (7)
Others (inspector, lecturer)	7 (8)
Location	
City	65 (75)
Province	22 (25)
Service level	
Tertiary hospital	30 (34)
Secondary hospital	37 (43)
Others (MoH, HRISRU, SIA, university, college)	20 (23)
Work experience	
Total years worked	17 (0.2 - 46)*
Years worked in current position	8 (0.2 - 29)*

Note: * Mean and range in years.

### Challenges and barriers to the formulation of infection control policy

#### There is a lack of evidence that HCAI is important in Mongolian hospitals

The majority of the participants claimed that, due to a lack of good statistics and evidence, infection control receives less attention and consequently gets few resources. The MoH officials and some hospitals administrators explained that “*feeling that infection control is important is not enough to allocate limited resources”.*

“I haven’t seen any reports on the burden of HCAIs in Mongolian hospitals. I remember only one number −0.05% in the annual statistics book which is very low”[MoH]

“Generally, I feel that there is a mess..and something has to be done [in infection control]…but to make a decision we need evidence, statistics which we don’t have. …In recent years, the health budget has been increasing rapidly. Therefore, it is not that difficult to fund activities. Now, there is money, but it is limited and [we] only need to allocate [the budget] wisely, which means we must carefully choose the really important activities… To choose the right one we should look at evidence. We can’t always spend money based on our feeling that is important” [MoH]

“It is very difficult to allocate resources to activities without justification,…For example, since last year we have been spending money for disposable syringe boxes. And now after18 months, I don’t have any idea what effect is given by this money. Actually, it wasn’t a small amount of money. We spent money but there are no measured outcomes.”[Hospital director].

#### The MoH lacks experts in modern infection control

Participants from the MoH and ICPs perceived that they have difficulty in updating guidelines and implementing surveillance and control measures due to a lack of technical knowledge in modern infection control. Key informants from the MoH and HRISRU explained that, in 2007 the MoH faced a problem of finding technical experts to update existing infection control guidelines. A doctor who recently completed a degree in infection control abroad was assigned to lead the working group, but the team was unable to fully amend the guidelines due to limited technical knowledge in some specific areas of infection control. Some issues such as developing laboratory-based surveillance and surveillance for antibiotic resistance were omitted. Participants from the SIA voiced the opinion that, since the transition to democracy, the MoH has been employing many non-specialized professionals in positions that require technical expertise and therefore many programmes are not implemented fully.

*“All our infection control people are graduates of the old Russian program. There is a shortage of manpower trained in modern or western infection control* [HRISRU]*.*

*Last year* [2007], *we had difficulties to find a person who can lead the committee to update guideline. Luckily, we found someone who just completed* [a degree] *in infection control … …but the working group couldn’t finish all the chapters of the guideline” [MoH].*

#### Punitive attitudes are existing in infection control

Study participants perceived that many officials believe that *“HCAI is a serious violation of quality of care that should result in the application of strict administrative measures*” and, therefore, the HCAI rate was included in the targeted performance evaluation in 1997 and, since then, hospitals and professionals who reported HCAI cases have been penalised. Participants believe that this strict control and penalization as a response to reported cases has led to dishonest reporting of infection control data.

“It is just rumour… people say that big hospitals don’t report their cases in order to avoid trouble. [ICP].

“According to the law, it’s our responsibility, and we do apply administrative sanctions.” [SIA].

#### There is no focal point at the MoH

According to the participants, the MoH has no staff in-charge of HCAI control policy and, therefore, infection control issues (related to HIV, blood transfusion, sterilization of equipment, etc.) are solved independently in different ministry divisions. The HRISRU staff claimed that for infection control issues they have to “*approach different ministerial people from different divisions*”. Officials from the MoH explained that *“If there is no internal person who brings issues to attention, then problems remain unsolved”* and therefore infection control remains neglected.

“At the MoH, infection control issues are solved in separate clinical divisions. Therefore, some divisional infection control plans are not well synchronized with other divisional plans. In the new MoH structure, I have suggested to create a new position which will be in-charge for coordinating infection control policy and plans at the national level” [MoH].

“As I am in charge of maternity health, I only coordinate infection prevention and control activities for newborns and their mothers” [MoH].

#### The infection control committees at both national and hospital levels are not functioning well

Study respondents explained that the National Committee for the Prevention and Control of Hospital Acquired Infections that was formed at the MoH from various organizations and expert representatives has never held a meeting since its establishment in 1997. They claimed that this was because the committee was “*too big”* to meet regularly; the committee’s terms of reference were not clear, particularly in terms of when the meetings should be called and by whom; and the committee has no budget to sustain regular activity. Participants from the HRISRU explained that because the committee has not been active, the MoH has amended the composition of the committee twice in the last 11 years. Due to the last amendment [May 2008], the committee came to consist of only three people from one tertiary hospital where the HRISRU is based, with none from the MoH. According to the ICPs, the situation passed from one extreme (too big) to the other: “*tiny and powerless*”.

“*There is a committee at the MoH but I don’t know if they meet …* [MoH]."

“*I don’t remember when the [national] committee held a meeting. Perhaps, not once since its establishment in 1997*” [HRISRU]."

Hospital ICPs claimed that in many hospitals, the Hospital Infection Control Committee (HICC) “*exists only on paper*”. HICCs do not hold meetings as often as guidelines recommend because the committee members are not willing to participate. Some hospital managers acknowledged that, to overcome audit from the Inspection Agency, ICPs are sometimes advised to write false minutes to show that HICC meets regularly. This is a form of gaming.

“*When I call them for a meeting, everyone becomes busy and we couldn’t meet this year …occasionally, I write fake meeting minutes to show inspectors…”[ICP]*

“*I chair 13 to 14 committees at our hospital… I can’t attend all of them” [Hospital manager]*

#### The Health Related Infection Surveillance and Research Unit has little power or capacity

The HRISRU staff claimed that they face difficulties in managing infection control programs at the national level because both MoH and hospitals are not supportive. According to them, many HRISRU suggestions sent to them were not absorbed or implemented. At the same time, they complained that none of the six HRISRU staff had completed any formal training in infection control and they experience challenges in their everyday work.

“*We have no support from both the “top” and surrounding people… 6 people share two computers… We don’t have a budget for travel, thus we can’t reach province hospitals…We don’t know what to do and how to do it. However, we do train others* [HRISRU]."

“These people [at the HRISRU] are graduates of the old Russian time. They need to learn modern infection control [MoH]

#### The current health financing system doesn’t account for the financial burden of HCAIs

Many hospital managers highlighted the importance of building financial mechanisms in Mongolia to motivate infection prevention. They criticized the MoH and the Health Insurance Fund for not establishing structures and mechanisms that produce evidence on how much money is “*wasted*” on treatment of HCAI in Mongolian hospitals and claimed that they “*don’t care because it is public money*”. Respondents from Health Insurance Fund explained that because the current health insurance system has no access to hospital adverse event data, they can’t use any incentives to make hospitals motivated in reducing costs for HCAI.

*“It’s public money, who cares…? Hospitals don’t care how much they spend for antibiotics and doctors just bombard patients with antibiotics”*[Hospital director]"

*“At the moment, our* [Health Insurance] *system cannot estimate the financial burden of HCAIs”* [Health Insurance Fund]."

### Challenges and barriers to the implementation of infection control policy

#### Resource allocation decisions are often made by non- medical professionals

Study participants claimed that MoH officials, mainly those who have a non-medical background, tend to cut resources planned for infection control activities. Participants claimed that the “*situation is worse*” in non-MoH hospitals [hospitals for defence, police and transport sectors are managed by their respective Ministries], where all decisions are made by non-medical ministerial officials.

Last year, our [hospital] budget for syringe boxes was cut by the financial people at the Ministry of Health and later in the Ministry of Finance. I was blamed… for not meeting these people and explaining properly for what and why this money was planned [ICP]

*“It is extremely difficult to convince people at the ‘top’ because they are non-medical”* [Military hospital doctor]"

Hospital ICPs also explained that, at the hospital level, many infection control decisions are made by finance or human resource managers, or engineers and, thus, infection control receives a low priority.

*“Are you really going to throw this money to garbage?” asked our hospital financial officer about the budget proposal for syringe boxes”* [ICP]"

#### ICPs are distracted by administrative tasks

Hospital ICPs explained that because the HICC has not functioned well and other colleagues are not cooperative, ICPs are alone in the hospital in their efforts to deal with all sorts of infection control issues. They complained that hospital administrators give ICPs a variety of tasks that are not fully related to infection control. This was supported by the managers of some hospitals that, with the intention of empowering ICPs, made them the head of their Infection Control Department, which includes units for cleaning and housekeeping, and sterilization and disinfection. This made ICPs more involved in administrative work rather than infection prevention and control.

*Most of my time I spend doing various administrative tasks plus dealing with waste disposal, cleaning, sterilization, sewage problems and even fighting against cockroaches and mice*” [Hospital ICP]"

“*Managing a department of over 20 staff is a high workload*…*The intention was to give her [ICP] more power but I suspect that the current structure distracts her [ICP] from infection control tasks*” [Hospital director]"

#### The laboratory system has limited capacity to support surveillance of HCAI

All group participants expressed concerns about the limited capacity of hospital laboratories. Laboratory physicians explained that due to outdated equipment and limited supply of consumables, anaerobic and viral cultures are not performed; bacteria are not identified to species level; some hospitals restrict the number of specimens that can be processed daily; and approximately half of hospital laboratory resources are spent on analysis of environmental swabs. Participants from district hospitals explained that none of six urban district hospitals of Ulaanbaatar have a microbiological laboratory.

*“Most of our lab equipment is from the 60s and 70s… often we face shortages of reagents and disks… we only do bacteriology tests …it is rare for anaerobic bacteria…we don’t identify bacteria to species level. There are no national standards for laboratory methods… we have a very high workload”* [Tertiary hospital lab physician]"

“*We don’t have a bacteriology lab at all …like all other district hospitals we send specimens to tertiary or private hospital labs.*” [District hospital ICP]"

Additionally, doctors participating in our study raised two concerns. First, because testing methods are not standardized across all hospitals, doctors commonly request that tests are repeated at their hospital adding more load to the laboratory. Secondly, some doctors, especially surgeons, prefer to prescribe antibiotics empirically because patients tend to be discharged around the time that antibiotic susceptibility test results are sent to the ward.

*“I can’t order bacteriology tests… because test results come on the day of the patient’s discharge or a day before, it’s just useless…”* [Surgeon]"

#### Antibiotic usage is not well regulated

All group participants recognised that antibiotic usage is not well regulated in Mongolia. While MoH officials were concerned more about the quality of antibiotics and the sale of antibiotics by the pharmacy without a physician prescription, hospital managers and ICPs worried about poor implementation of antibiotic guidelines by doctors which resulted in overuse of antibiotics. Doctors disagreed with this statement by complaining that tertiary hospital laboratories are not capable of providing fast and reliable susceptibility testing, no metropolitan district hospitals have a bacteriology laboratory, and hospitals provide cheap, simple and often fake antibiotics. Therefore, when patients take strong antibiotics prior to admission to hospital, doctors have to require patients to bring stronger antibiotics from the community pharmacy which is contradictory to hospital antibiotic guidelines.

“Every patient is treated with antibiotics, even children with viral diarrhoea… There are many fake drugs in the market…Doctors complain that some antibiotics have no effect, …and presumably that’s why our doctors tend to prescribe the strongest and most expensive one” [Hospital director]

“Patients take strong antibiotics prior to admission to hospital… and, at the hospital, they [patients] will need stronger antibiotics” [Doctor]

“Bacteria became more resistant… we need different antibiotics but the hospital supplies the same cheap antibiotics every year” [Doctor]

#### Hand-hygiene compliance is low

All group participants perceived that hand-hygiene compliance among health professionals of Mongolia is low. While participants from province and district hospitals reasoned it is mainly due to unavailability of hot water and sinks and a poor supply of soap, participants from urban tertiary hospitals claimed that it is because of poor supply of alcohol based hand sanitizers, skin care products and high workload of health professionals. Although many doctors and nurses complained about skin dryness and irritation, hospitals managers and ICPs noted that skin care products are not supplied in any Mongolian hospitals. Hospital ICPs also wonder that, despite most hospitals conducting staff hand-hygiene training once or twice a year, hand-hygiene compliance remained poor. According to them to improve hand-hygiene training they need well-designed training materials, posters and reminders. Hand–hygiene compliance level is not monitored in any hospitals.

“People know that they should wash their hands, but they don’t. It’s poor accountability…We are planning to install camera systems in hospital delivery rooms to monitor hand washing”[MoH].

*“Everybody knows when and how to wash their hands but they don’t* ”[Hospital manager]"

“*My skin often gets dry… and I buy hand cream..because the hospital doesn’t provide it” [Doctor]*

#### Poor disinfection and sterilization

Participants from the HRISRU explained that no one is in charge of disinfection and sterilization at the MoH and there are no sanitation experts in the HRISRU team. They are confused about whether or not they are responsible for disinfection and sterilization. According to them, standards and guidelines for disinfection and sterilization have not been updated and hospital equipment remains obsolete. Hospital managers were sceptical about the quality of the few medical disinfectants and antiseptics available in Mongolia and doctors were concerned about the way disinfectants were used. They explained that hospitals don’t monitor levels of active compounds in the disinfectants. Hospitals managers said that they face a severe shortage of staff in charge of disinfection and sterilization, because they have the lowest salary in the health sector, and at remote clinics they have to hire untrained personnel to operate autoclaves. Additionally, ICPs from HRISRU explained that they face challenges to control disinfection and sterilization in private hospitals, which use various equipment and liquids bought from local markets.

“It [disinfection and sterilization]is the most unattended area of infection control. What we do is just replace a few autoclaves in hospitals and that is it. We need to do a lot in this area”[MoH]

*“We are not sure who should manage this issue”* [HRISRU staff]"

“*Our hospital has two BK-75 autoclaves [made in the 1970s in Russia]. They lose pressure, often break and we hardly ever find spare parts”* [Hospital ICP]"

“*We use chloramines everywhere but nobody monitors whether these disinfectants are capable of killing pathogens*” [Surgeon]."

#### Poor implementation of occupational health programmes

Based on some hospital studies, study participants claimed that there is high level of occupational exposures and infections among health professionals of Mongolia. However, they explained that, due to budget limitations, personal protective equipment (PPEs) such as masks, gowns and gloves are supplied with occasional interruptions and no vaccination and treatment is provided to health professionals. While MoH officials announced that, since March 2008, a new policy required hospitals to provide syringe boxes in all clinical areas to reduce sharps injuries and spill exposures, ICPs explained that its implementation has been delayed due to severe budget shortages and as a result many hospitals use handmade boxes that are not needle-stick and liquid-spill safe. According to the HRISRU, hospitals were advised to start occupational exposure registration in 2008 but data are not yet collected at national level.

“…60-80% of our surgeons are diagnosed with hepatitis B and C virus infections… but there is no money for treatment…and vaccination” [Hospital director]

“Now, I have positive tests for chronic hepatitis B. I was young and healthy when I started my work here in this hospital 25 years ago…But I don’t know when I was actually infected with this hepatitis infection. Hospital annual health check-ups started recently [early 2000]”[Surgeon]

“As syringe boxes are expensive, our nurses make them from ordinary boxes” [ICP]

#### Poor hospital infection control knowledge among health professionals

All study group participants acknowledged their poor knowledge of infection control. Infection control is not well taught at the undergraduate level. Hospital ICPs complained that the current 5-year university programme they have completed is designed for hygiene -inspectors and they had to learn hospital infection control *“from scratch*”. Doctors said that they “*don’t remember*” what they were taught on infection control during their undergraduate studies. Study participants suggested urgently updating the current Mongolian university and college curriculum. Recently, the Health Sciences University of Mongolia has established a 6-month post-graduate course for ICPs but due to a shortage of lecturers the course is managed by HRISRU staff.

Copies exist of only one infection control book in Mongolia which was translated by the HRISRU in 2002. Participants claimed that the internet is the main source of new information but access to the internet, a lack of subscriptions to infection control journals and language barriers limit the ability of health professionals to update their knowledge. Infection control does not seem to be a favourite subject for research in Mongolia. According to HRISRU staff, only three masters and one PhD student graduated in infection control in the last two decades. They explained that professional associations in infection control are not well established in Mongolia, mainly due to financial difficulties, and a lack of expertise and support from the government

*“At the medical university I trained to be a hygienist. Most of our classmates now work as hygiene inspectors. It was quite challenging for me to decide to work at the hospital. When I started work, I had to learn* [IC] *from scratch from our colleagues”* [Hospital ICP]"

*“ I don’t remember what I was taught at Uni on infection control”* [Doctor]"

“*It is common that ICP can’t answer questions from staff and I had to manage to not embarrass her* [the ICP] *in front of their colleagues…”*[Hospital director]"

*“Those doctors and nurses who went for overseas training or those who have good English quite often bring me information about new modern hospital infection prevention methods… and disinfectants. Every time they explain something to me, I felt that was I supposed to be teaching them, not them teaching me.”* [Hospital ICP]"

## Discussion

Sub-optimal infection control constitutes an important healthcare problem in Mongolia. This study identified a large number barriers and challenges that hinder effective infection control in Mongolia. Barriers to the formulation of infection control policy include: a lack of valid infection control statistics and experts; the absence of a focal point at the MoH; a poorly functioning national committee; and a lack of power and capacity of the national management unit. Barriers to the implementation of infection control policy and plans include: poor infection control education of health professionals; limited laboratory capacity; inappropriate use of antibiotics; low compliance with hand hygiene; poor disinfection and sterilization; and poor implementation of occupational health programmes. To better interpret these findings, reported barriers and challenges are grouped into the following groups. These are:

### Barriers and challenges related to poor funding

A lack of resources for infection control is a major challenge for resource-limited developing countries [3,7]. In Mongolia, scarce healthcare resources may explain some major infection control challenges such as poor laboratory capacity, rudimentary sterilization equipment, fake antibiotics and disinfectants and low salaries for health professionals. The following factors might contribute to the lack of resources for infection control programs:

Overall investment allocated for healthcare is limited. While industrialized and developed countries spend 8 to 16% of Gross Domestic Product (GDP) for healthcare in 2010 [16], like other resource-limited countries, the Government of Mongolia spends only 3.0% of (GDP) on healthcare (there was a decrease from 4.9% to 3.0% since 2001) [17].

Trade off with vital clinical areas is often made. Our study findings suggest that when allocating resources, policymakers, managers and practitioners in Mongolia often have to choose between infection control and vital clinical areas such as drugs, anaesthetics, laboratory consumables or other hospital core survival expenses including salary and heating. Therefore, infection control activities often remain under resourced.

A low priority is given to infection control. The current official infection control statistics of Mongolia, which show the annual prevalence of HCAI being 0.01–0.05%, is the only available report for health professionals of Mongolia [18]. Our recent studies showed that these MoH statistics were a drastic under-estimate of true burden of HCAIs in Mongolian hospitals, masking infection control problems from decision makers [15,19]. Yet, other statistics on hand-hygiene compliance, occupational exposure and infection levels, antibiotic usage and resistance, and financial burden of HCAIs are not available for decision makers. Hence, an absence of complete and valid statistics and other supporting evidence may cause difficulty for decision makers when justifying resources for infection prevention activities. Therefore, it is important for local ICPs to produce evidence for decision makers, so that infection control receives more attention and resources.

Wasteful practices widely exist. Study participants described many practices that are considered not cost-effective elsewhere [20]. This includes practices such as a half of the hospital laboratory resources spent on routine environmental swabs while some hospitals restrict the number of specimens that can be processed daily; antibiotic susceptibility test results are not used for prescriptions because test results come late and excessive amounts of antibiotics are used for prophylaxis. These practices divert resources from more cost-effective practices leading to more severe resource shortages. Damani suggests replacing these practices with low-cost measures including training of hospital staff, promoting hand hygiene, staff immunization, etc. [20].

### Barriers and challenges related to suboptimal knowledge and attitude of health professionals

A lack of expertise and knowledge on modern infection control is a common challenge in developing countries [6,7,9]. In Mongolia, infection control is not well taught both at the under- and post-graduate levels of medical education. This may explain the following:

ICPs are not confident in developing plans, establishing surveillance, updating guidelines and leading other healthcare workers toward building modern infection control systems;

Healthcare workers are not well aware of the importance of infection control and they are not supportive of infection control initiatives;

The traditional approach of policy makers to infection control, which is characterised by the excessive penalization of reported HCAI cases, led to various types of gaming including excessive antibiotic prophylaxis [15].

Hospitals have limited access to internet and healthcare professionals lack updated clinical guidelines and books in the local language;

As clear policies and active support for training appear to be vital determinants of effective practice and successful change [21-23], Mongolia needs to develop infection control education policy together with organisational mechanisms for supporting continuous professional development. Meanwhile, the International Nosocomial Infection Control Consortium (INICC) collects data from 18 limited-resource countries and provides guidance in improving infection control measures at a local level [24]. Similar initiatives are implemented in Europe [25,26].

### Barriers and challenges related to inadequate management

The review by Griffith et al. (2009) highlighted that positive proactive leadership, support and presence of senior leaders, team commitment, and clear boundaries of roles and responsibilities are prerequisites for effective action to control infections [27]. Our study revealed the following:

Weak leadership at the policy level has resulted in failure to implement the national plan to establish surveillance for certain HCAIs;

National and hospital level infection control committees lack committed professionals and as a results these committees do not function well;

Infection control regulations, standards and guidelines lack clear descriptions of the roles and responsibilities of individual professionals, committees and organizations. Therefore, National committee members were not sure about when and how they should meet; HRISRU were not sure whether they are responsible for disinfection and sterilization; the MoH had no person in charge of infection control, and hospital ICPs are distracted by administrative tasks.

These findings clearly show that there is a considerable need for raising issues of commitment, accountability and ownership in Mongolia so that: ICPs are enabled to lead others working across various disciplines, units and management levels; clinicians clearly understand and fully implement their roles and responsibilities; and senior level managers enable and support infection control initiatives. To help ICPs overcome local barriers, the WHO “Clean Care is Safer Care” campaign that focuses on hand hygiene received pledges of commitment to make progress from over 120 ministries of health [28]. Although this strategy is implemented on a voluntary basis, more countries are assigning up to membership of these initiatives, building new benchmarks and peer pressure for their lagging neighbours. In addition, increasing public awareness will have a significant impact on accelerating government plans for safety and quality in health care [29].

Similar to our findings, poor infrastructure, insufficient equipment, understaffing, paucity of knowledge, inappropriate use of antibiotics, and scarcity of local and national guidelines and policies were reported as common barriers to effective implementation of infection control in developing countries [7,8]. In response, simple, low-cost, high-impact infection control strategies, such as hand-hygiene improvement programmes and simplified process surveillance have been suggested by several authors [20,30,31]. However, without necessary training of key personnel, administrative support and provision of necessary resources, it is impossible to implement these recommendations [32-34]. Therefore, actions with logistical, educational and management components that are specific to local circumstances need to be designed and implemented in Mongolia. It is worthwhile to seek support from international professional organizations such as INICC [35].

This study has the following two main limitations. Firstly, due to resource constraints, the data translation, transcription, coding and quotation selection processes were performed by a single researcher (B-E.I) rather than by two or more investigators. However, the constant iterative discussions within the research team regarding fieldwork experience and analysis maintained the validity of our findings. Secondly, the study examined issues from the participants’ perceptions and there is an obvious need to complement and extend the work presented with large scale quantitative and mixed-method investigations that can provide data and findings on a national scale and with statistical significance. More detailed research will be needed in each area of infection control, including hand hygiene, disinfection sterilization, occupational health, waste management, infection control education and ICP workload, to fully comprehend all of the issues related to their implementation. Moreover, it is important to fully understand interactions and interrelationships of the existing aforementioned contributory factors to poor infection control practice in Mongolia. Root cause or system analysis methods could provide a suitable framework for further research on infection control in Mongolia.

## Conclusions

The availability of infection control policy and guidelines, and provision of specific recommendations have not assured effective implementation of infection control programmes in Mongolia. The current infection control system in Mongolia is likely to remain ineffective unless the underlying barriers and challenges are adequately addressed. The nature of these barriers and challenges is complex and require further appropriate assessments which enable the implementation of multifactorial strategies to improve infection control.

## Competing interests

All authors report no conflicts of interest relevant to this article.

## Authors’ contributions

B-EI, ACAC, JA, AM and MW developed the research question and designed the study. B-EI collected data and drafted the manuscript. B-EI, ACAC and JA performed the thematic analysis. B-EI, ACAC, JA, AM and MW finalized the manuscript and MW found a source for manuscript payment. All authors have read and approved the present manuscript.

## Pre-publication history

The pre-publication history for this paper can be accessed here:

http://www.biomedcentral.com/1471-2334/12/170/prepub
